# Motivational Interviewing for Workers with Disabling Musculoskeletal Disorders: Results of a Cluster Randomized Control Trial

**DOI:** 10.1007/s10926-017-9712-3

**Published:** 2017-05-26

**Authors:** Joanne Park, Shaniff Esmail, Fahreen Rayani, Colleen M. Norris, Douglas P. Gross

**Affiliations:** 1 Department of Occupational Therapy, University of Alberta, 3-48 Corbett Hall, Edmonton, AB T6G 2G4 Canada; 2 Workers’ Compensation Board of Alberta Millard Health, Edmonton, AB Canada; 3 Department of Occupational Therapy, University of Alberta, Edmonton, AB Canada; 4 Workers’ Compensation Board of Alberta, Edmonton, AB Canada; 5 Faculty of Nursing/Public Health, School of/Medicine & Dentistry Medicine/Surgery, University of Alberta, Edmonton, AB Canada; 6 Department of Physical Therapy, University of Alberta, Edmonton, Alberta Canada

**Keywords:** Motivational Interviewing, Musculoskeletal, Return-to-work, Rehabilitation, Workers’ Compensation

## Abstract

*Purpose* Although functional restoration programs appear effective in assisting injured workers to return-to-work (RTW) after a work related musculoskeletal (MSK) disorder, the addition of Motivational Interviewing (MI) to these programs may result in higher RTW. *Methods* We conducted a cluster randomized controlled trial with claimants attending an occupational rehabilitation facility from November 17, 2014 to June 30, 2015. Six clinicians provided MI in addition to the standard functional restoration program and formed an intervention group. Six clinicians continued to provide the standard functional restoration program based on graded activity, therapeutic exercise, and workplace accommodations. Independent* t* tests and chi square analysis were used to compare groups. Multivariable logistic regression was used to obtain the odds ratio of claimants’ confirmed RTW status at time of program discharge. *Results* 728 workers’ compensation claimants with MSK disorders were entered into 1 of 12 therapist clusters (MI group = 367, control group = 361). Claimants were predominantly employed (72.7%), males (63.2%), with moderate levels of pain and disability (mean pain VAS = 5.0/10 and mean Pain Disability Index = 48/70). Claimants were stratified based on job attachment status. The proportion of successful RTW at program discharge was 12.1% higher for unemployed workers in the intervention group (intervention group 21.6 vs. 9.5% in control, p = 0.03) and 3.0% higher for job attached workers compared to the control group (intervention group 97.1 vs. 94.1% in control, p = 0.10). Adherence to MI was mixed, but RTW was significantly higher among MI-adherent clinicians. The odds ratio for unemployed claimants was 2.64 (0.69–10.14) and 2.50 (0.68–9.14) for employed claimants after adjusting for age, sex, pain intensity, perceived disability, and therapist cluster. *Conclusion* MI in addition to routine functional restoration is more effective than routine functional restoration program alone in improving RTW among workers with disabling MSK disorders.

## Introduction

Musculoskeletal (MSK) disorders result in substantial direct costs to health care systems and even larger indirect losses on productivity [1]. Although there is a high prevalence of MSK disorders in industrialized countries, a disproportionate share of the cost is related to a small proportion of cases associated with chronic pain [1, 2]. Evidence indicates that incapacity and chronic work disability are behaviours that are often the result of psychosocial factors [3]. Although recent evidence indicates a possible decrease in the total number of workers’ compensation claims over the years, the cost of claims and number of paid days for compensation has increased [2]. The majority of injured workers (approximately 80–85%) return-to-work (RTW) quickly and without complications; however, the remaining 15–20%, experience long periods of work disability [4]. Additionally, their disability may be coupled with personal, emotional and/or work related issues that contribute to their delay in returning to work [4, 5]. It is estimated that 15–20% of workers with chronic work disability account for approximately 70% of the cost of work related disorders [6]. Physical limitations coupled with psychosocial issues influencing the worker’s behaviour may be contributing factors associated with the increase in the number of paid compensation days, an increase in claims cost and ultimately a delay in RTW.

The importance of a timely RTW after a medical absence is critical in the recovery process and ensuring employees RTW after an injury. This is especially important for injured workers who have been absent from work for 3 months or longer as the probability of returning to work decreases by ~50% after 12 weeks [4]. The percentage of those returning to work significantly decreases by 24 weeks with only a ~20% RTW rate and by 48 weeks only ~2% of employees RTW post physical/psychological illness or injury [4]. Employees who have not returned to work within 3 months of their injury have a higher probability of following a course of chronic work disability [6].

Health care costs, absenteeism, presenteeism, and productivity are all either directly or indirectly linked to behaviour-related health practices [7, 8]. In addition, behavioural choices such as physical activity and substance abuse are important influences on health outcomes like morbidity and mortality [7]. Health coaching is a behavioural intervention that has gained recognition in public health, health promotion, and disease management for its capacity to address several behaviours, health risks, and self-management of illness using a cost effective method [7]. Health coaching is an approach used by providers that facilitates clients in shifting and/or changing their behaviours in order to improve their health, quality of life or establishing and achieving their health promotion goals [9]. Motivational Interviewing (MI) is a counselling method often incorporated into the process of health coaching and frequently outperforms conventional advice-giving treatments for a wide range of behavior related problems and diseases including drug and alcohol abuse, smoking cessation, weight loss programs, increasing physical activity, and medication and diabetes management [10–13].

MI is defined as being client-centered in nature but also directive in guiding clients towards behavioural change by assisting them in identifying and resolving ambivalence [14]. Ambivalence has been defined as having conflicting opinions or feelings about something, and can be an important barrier to behavioural change [15]. The philosophy of MI suggests individuals approach change at varying levels of readiness and the role of the assisting professional is to help increase client awareness in terms of implications for change [16]. MI is a method of assisting clients to identify and react to their current or potential issues and is viewed to be largely useful with individuals who are disinclined or ambivalent about changing their behaviour [13]. The strategies of MI are more persuasive and supportive compared to traditional counseling methods that tend to be more coercive and argumentative; therefore, the goal of MI is to increase the individual’s intrinsic motivation resulting in a change that occurs from within instead of being forced upon from without [13].

Client motivation is essential for successful rehabilitation. Research has shown that client motivation may be influenced by a variety of factors including the individual and environment as well as the rehabilitation process itself [4, 17]. From a clinical perspective, the identification of modifiable risk factors affecting work disability, such as motivation and ambivalence, could assist in establishing targeted interventions that can avert the development of chronic work disability [6]. Ambivalence regarding RTW occurs when claimants experience doubt, uncertainty, and/or contradictory ideas regarding their ability to perform work. This uncertainty can reduce motivation and prevent claimants from looking for work or testing their abilities through an actual RTW trial. MI may be an effective tool in addressing both MSK disorders and psychosocial/behavioural barriers that limit a timely RTW for the 15–20% of workers who experience greater than normal complications with their RTW process.

MI is efficacious in reducing maladaptive behaviours and promoting adaptive behaviours in a broad range of situations where behaviour change is the focus, but has not been tested in the injured worker population [13]. Characteristics of MI techniques that complement its suitability for potential use in work rehabilitation programs focusing on behavioural change include: (1) its effectiveness with clients who are ambivalent or reluctant to change their behaviour; (2) it is efficacious even in small treatment quantities; (3) it can be applied across age, gender, cultural and socioeconomic statuses; and (4) it fits well in combination with conventional interventions and programs [18].

## Objectives and Study Hypothesis

We evaluated the effectiveness of MI in a population of injured workers receiving workers’ compensation and undergoing work rehabilitation. We hypothesized that MI in addition to a standard functional restoration program would lead to a higher proportion of successful RTW among workers without jobs to return to. We anticipated that unemployed injured workers (not job attached) would be more ambivalent about returning to work due to uncertainty about their employment.

## Methods

### Study Design

In this cluster randomized controlled trial, a sample of 728 workers’ compensation claimants with MSK disorders were entered into 1 of 12 therapist clusters (MI group = 367, control group = 361). This is a cluster RCT as clinicians in the rehabilitation programs were randomized as opposed to individual claimants. The intervention group included six clinicians who were formally trained in MI prior to commencement of the study and provided MI intervention in addition to a standard functional restoration program. The control group clinicians continued to provide the standard functional restoration program based on graded activity, therapeutic exercise, and workplace accommodations. The University of Alberta’s Health Research Ethics Board approved this project.

The study took place at Millard Health, the primary occupational rehabilitation service provider for the Workers’ Compensation Board (WCB) Alberta, Canada. All data were obtained from WCB Alberta clinical and administrative databases. There were no changes to methods after commencement of the study; however, one clinician in both the intervention and control groups were excluded prior to the start of the study as they either changed departments or were no longer an employee of Millard Health/WCB Alberta.

### Sample

All 728 claimants were Alberta workers who were injured at work. Claimants were included in the study if they had active workers’ compensation claims for a MSK disorder and were participating in a RTW program between November 17, 2014 and June 30, 2015. Sample size was estimated for conducting a logistic regression testing the effect of group status while controlling for nine potential confounders using a small inflation factor (intracluster correlation = 0.05). We aimed for and achieved over 100 claimants per intervention group.

Claimants were excluded from the study if they were under the age of 18, referred for surgery during the time of their RTW program, had a traumatic brain injury or traumatic psychological injury (these took precedence over MSK disorders), or could not read, write or speak English independently (i.e. those requiring an interpreter were excluded). Claimants were removed from the study if they had co-morbid conditions interfering with their rehabilitation, discharged due to non-compensable medical reasons or non-compliance with rehabilitation, or attended their RTW program for less than 5 days (i.e. did not actually participate in rehabilitation).

### Procedure

Data collection was completed as part of the routine rehabilitation process, with the MI intervention added as a component of standard care for claimants in the intervention group. This permitted us to avoid disrupting service delivery at the facility while also providing a pragmatic context for the clinical trial.

Instead of randomly allocating individual claimants to study groups, we randomized the primary clinicians (clinicians responsible for overseeing the RTW program). Claimants were therefore considered to have entered ‘clusters’ of individuals treated by the same clinician. This process has successfully been used in previous research studies conducted at the facility [19–21]. All primary clinicians were randomly allocated to one of two groups, an intervention group or a standard care control group. To form the two groups, two of the researchers (DG and JP) generated the random allocation using a computerized random number generator (http://www.random.org). Clinicians were assigned a number with odd numbers indicating intervention group membership. The clinicians included occupational therapists and exercise therapists. The intervention group included four occupational therapists and two exercise therapist while the control group consisted of two occupational therapists and four exercise therapists. Clinicians in the intervention and control groups were generally in their 30s (intervention group average age 30 years; control group average age 35 years) and were female (intervention group = 6 females; control group = 5 females, 1 male).

Prior to beginning the trial, clinicians assigned to the intervention group were formally trained in the fundamental processes of MI by an experienced trainer and certified member of the Motivational Interviewing Network of Trainers (MINT). Intervention group clinicians added an MI approach to standard functional restoration programs while clinicians in the control group continued to provide a routine functional restoration program. Clinicians made all claimant level treatment and RTW decisions. Logistics involving admissions and treatment process at the occupational rehabilitation facility where the data collection occurred was not altered. The number of MI sessions provided depended on clinical judgement of the intervention clinician with recommended intervention duration of 30–60 min as per previous MI studies identified in a recent systematic review [13].

Due to the nature of work rehabilitation, neither clinicians nor patients were completely blind to group allocation. However, claimants were not aware of the study and were blinded to group membership. Additionally, outcome evaluation was performed in a blinded fashion by obtaining information on claims outcomes from WCB administrative databases. Comparisons were made on key outcomes between claimants seen by clinicians within the two groups.

## Intervention

### Motivational Interviewing

The clinical procedure of MI involves a conversation about change with the primary purpose of strengthening the client’s own motivation for change [14, 15]. The role of the MI practitioner is to evoke change talk within their clients, which is an expression of the client’s desires, reasons, ability, and need for change [14, 15]. The probability of behaviour change increases with verbalized intention and a detailed plan for implementation [18]. Evoking change talk using an empathetic and supportive approach reinforces the client’s motivation and commitment to change [14, 18].

Miller and Rollnick describe four central processes (engaging, focusing, evoking and planning) that form the flow of MI which was used by our intervention clinicians [15]. The first process is engaging, which is necessary to establish a helpful connection and working relationship. The process of engaging leads to focusing, the second central process, which is necessary to develop and maintain focus on a particular agenda. The third process in MI is evoking which involves eliciting the client’s motivations for change by having the individual voice their arguments for change [15]. The fourth process involved in MI is planning which includes developing commitment to change and formulating a specific action plan [15].

**Fig. 1:**
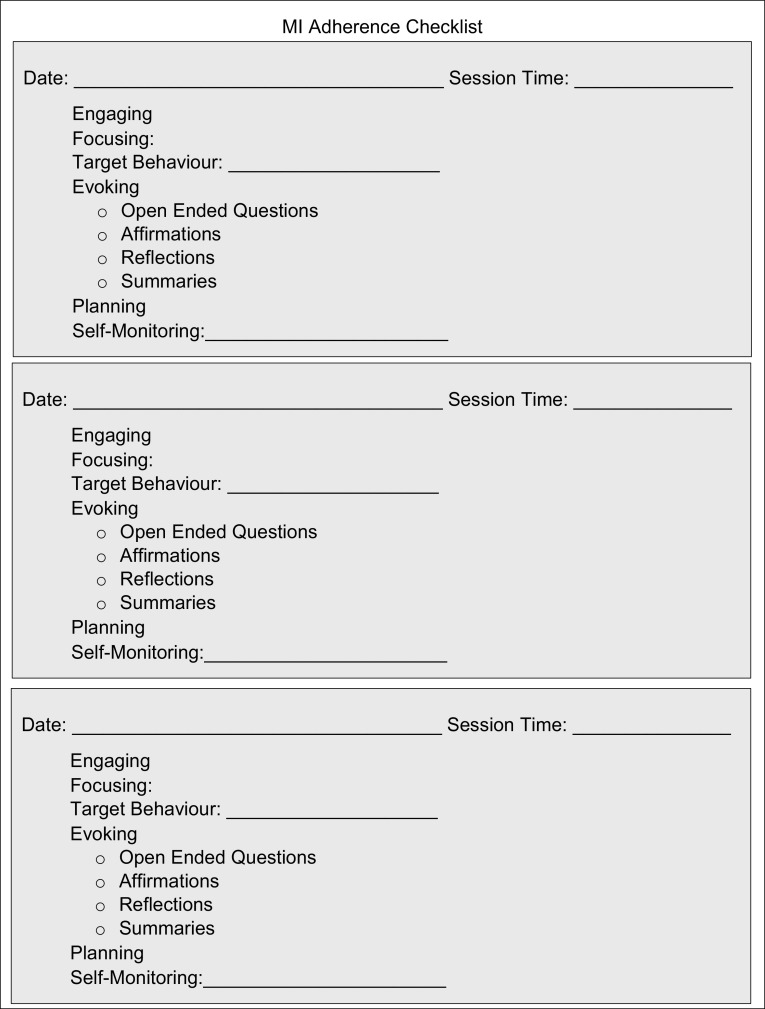
Motivational interviewing adherence checklist

During the intervention, clinicians made all treatment decisions for each claimant and individual MI sessions were tailored to the claimant and their identified target behaviour. MI has been reported to be more effective in those who are ambivalent about behaviour change [18]. Therefore, intervention clinicians were trained to listen for ambivalence in their client’s language to determine if their client was ambivalent and thus a candidate for MI. Intervention clinicians also completed a MI Adherence checklist (Fig. 1) after every MI session they completed with their clients. This was to ensure the clinicians were adhering to the fundamental processes used in MI and to track how many clients they completed MI with. In addition to this, formal monthly coaching sessions were completed for 1 h to promote MI skill development and provide an opportunity for the clinicians to ask questions about any difficulties they experienced using MI. Informal weekly meetings with the researchers were also completed to address any study questions.

### Functional Restoration Programs

The past 20 years has seen therapies and program dedicated entirely to treating occupational injuries existing as a range of programs including work hardening, occupational rehabilitation, industrial rehabilitation, and RTW programs [22]. Although the programs may have different names, their goals are fundamentally similar including restoring the physical abilities and functional tolerance of the injured worker in order to return them to gainful employment [22, 23]. RTW can be thought of as the process an injured worker undergoes in returning to work, their measureable fitness for work, and their vocational outcomes, including duration and/or extent of their inability to work as a result of their functional limitations [22–24].

Standards of practice, program parameters, and service delivery methods may vary depending on providers, the individual programs and the various disciplines involved in the RTW process, which can be influenced by the needs of the worker and employer, disability management practices, insurance policies, and the practice of the work rehabilitation programs [22, 25]. The standards of practice used for this study are the services offered at Millard Health in Alberta, Canada, the primary service provider for WCB Alberta. The current RTW services offered at Millard Health includes an interdisciplinary approach that focuses on improving physical and functional abilities, RTW planning, and individual counselling and educational workshops [26]. Referrals for services are based on claimant needs and the request for services from the claimant’s claim manager/adjudicator or employer. The goal of the RTW services offered at Millard Health is to assist with the gradual development of strengths and skills towards a timely and successful RTW, which often involves the participation of various stakeholders [26].

## Measures

### Intervention Variable

A dichotomous intervention variable was created indicating group allocation by identifying the intervention clinicians using WCB Alberta databases. The only difference between the intervention and control groups was the addition of MI to functional restoration programs for the intervention group, otherwise all other aspects of the RTW program delivery process were comparable across all claimants. Adherence to the MI protocol was also measured via tracking clinician completion of the MI adherence checklist. MI adherent clinicians were those who identified a target behaviour for the MI session and indicated on the adherence sheet that all the fundamental processes were completed during the session. Non-adherent clinicians did not complete the adherence sheet, meaning either they did not adhere to the MI protocol or else used MI but did not complete the required paperwork.

### Potential Confounders

We obtained information on several potential confounders including age, sex, gross annual salary, marital status, disability duration, and overall pain and disability scores to control for the possibility of unequal group formation. These variables were chosen based on previous studies examining injured workers within this setting, which showed some predictive value or theoretical rationale for considering the variables as potential confounders [19].

### Outcome Measure

The primary outcome was confirmed RTW status at time of program discharge, meaning that the claimant was returning to work after rehabilitation either with their date of accident employer or a new employer. If this is not the case, the claimant will either be discharged as fit for work (i.e. deemed capable of working but does not have a job to return to) or not RTW. RTW status was measured through percentage of confirmed RTW success and compared between the intervention and control groups. RTW status is an important claimant descriptor and claims measure used at the occupational rehabilitation facility where the data was collected. It informs clinicians and claim owners of the potential need for vocational services beyond the standard functional restoration program and/or the need for ongoing wage replacement benefits. Within the RTW rehabilitation program context, these measures are regularly used as indicators of employment and were available for 100% of our sample.

### Potential Harms

Review of MI literature did not identify any potential harmful effects [13, 15, 18]. In the event a claimant or clinician believed MI was causing adverse effects, psychologists and physicians were onsite to handle such circumstances. Any situations were to be followed-up and documented by the study team.

### Statistical Analysis

All data records were reviewed to determine if there were any issues with the data such as missing data, outliers and inclusion of excluded criteria. Descriptive statistics were then calculated. Independent *t* tests and chi square analysis were used to determine if a difference existed between groups for claimant characteristics and self-report pain and disability questionnaires. To determine if a clustering effect occurred, we examined differences across clusters on claimant characteristics. We also examined intracluster correlation using kappa for the dichotomous outcome measure. We then compared percentages of confirmed RTW success at time of program discharge, stratified based on job attachment status. Independent *t* test and chi square analysis was used to test our hypotheses and determine if there were any statistical differences between the MI intervention and control groups. Multivariable logistic regression was used to obtain the odds ratio of RTW at the time of program discharge while adjusting for potential confounders and cluster. An intention to treat analysis maintained the benefits of randomization of the clusters and a 0.05 alpha level was used to determine statistical significance. All analyses were conducted using IBM SPSS 23 (Armonk, New York).

## Results

### Claimant Characteristics

**Fig. 2:**
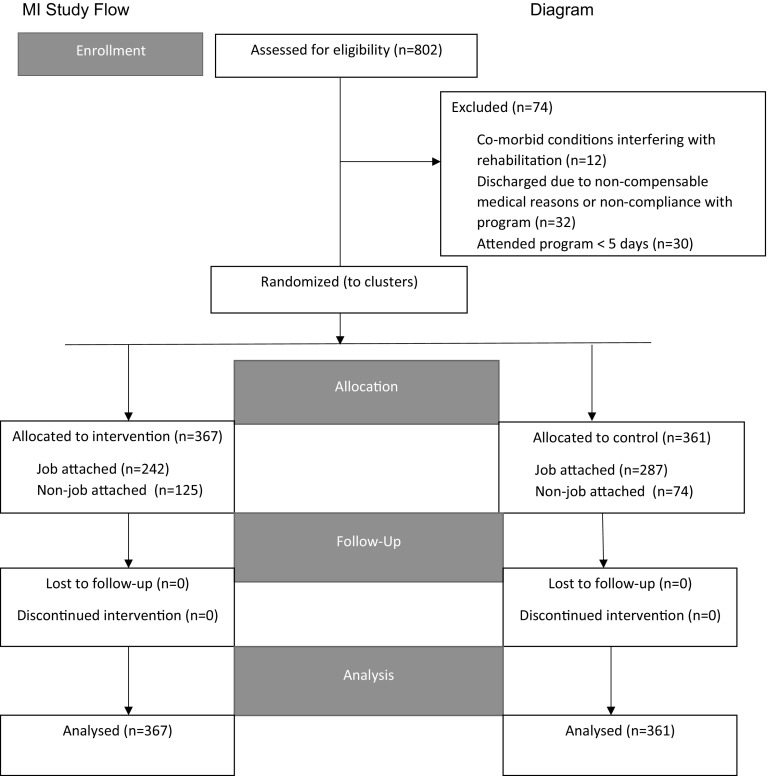
Study flow diagram

728 workers’ compensation claimants with MSK disorders were entered into 1 of 12 therapist clusters (MI group = 367, control group = 361). 74 claimants were excluded from the study due to medical reasons, non-compliance with their RTW program, or attendance in the RTW program for less than 5 days. For claimants who attended more than 1 RTW program during the study period, only data from the last completed RTW program was used since claimants at times are moved between programs until they are placed into the most appropriate program. A flow chart showing the enrollment, allocation, and analysis of claimants at each step of the study is shown in Fig. 2.

Table 1 presents a descriptive analysis of key claimant characteristics. Claimants were predominantly employed (72.7%), males (63.2%), in their mid 40–45 years (SD 12.2), married (39.6%), achieved a high school education, had an annual income of $59,800 CDN, with a disability duration of 233.7 days measured from date of accident to admission to RTW program, had moderate levels of pain and disability at program admission (mean pain VAS = 5.0/10 and mean Pain Disability Index = 48/70), and lower levels of pain and disability (mean pain VAS = 3.8/10 and mean Pain Disability Index = 35/70) at program discharge. No statistically significant differences were observed between claimants in the MI and control groups at program admission on claimant characteristics such as sex, marital status, income, education level, age, disability duration, pain intensity, or self-rated disability. A higher percentage of female claimants was observed in the control group versus the intervention group, however this was not statistically significant. There was a statistically significant difference in the percentage of claimants employed at time of referral to the RTW program between groups (p ≤ 0.01).

**Table 1:** Characteristics of claimants at referral for return-to-work program

	Entire sample (n = 728)	Intervention group (n = 367)	Control group (n = 361)
	Mean (SD) or %		
Employed at time of referral^a^	72.7	65.9	79.5
Age (years)	45 (12.2)	44 (12.0)	46 (12.3)
Sex (% male)	63.2	66.5	59.8
Marital status (%)			
Married	39.6	37.6	41.6
Single	29.4	30.2	28.5
Common-law	10.0	9.3	10.8
Widowed	1.4	1.4	1.4
Divorced	6.7	6.8	6.6
Separated	3.6	4.1	3.0
Not specified	9.3	10.6	8.0
Gross annual salary ($10K CDN)	59.8 (30.2)	61.4 (30.7)	58.1 (29.6)
Education level			
Grade 8 or less	3.8	3.8	3.9
Partial high school	14.0	13.9	14.1
High school diploma	24.7	23.4	26
Partial technical school	9.2	8.7	9.7
Technical diploma	19.1	20.4	17.7
Partial university	3.8	3.5	4.2
University degree	7.0	6.5	7.5
Not specified	18.3	19.6	16.9
Disability duration (days)	233.7 (688.0)	257.0 (721.8)	209.8 (652.1)
Admission Pain Disability Index (PDI, n = 719)	48.4 (21.0)	49.1 (20.2)	47.6 (2.2)
Discharge PDI (n = 624)	34.5 (23.4)	35.4 (23.5)	33.6 (2.3)
Admission Pain Visual Analogue Scale (VAS, n = 713)	5.0 (2.2)	5.1 (2.1)	4.9 (2.2)
Discharge VAS (n = 608)	3.8 (2.4)	3.9 (2.5)	3.7 (2.3)

*Statistically significant difference at p < 0.01

When testing for a clustering effect, the claimant characteristics that were statistically different (p < 0.05) across clusters were age, sex, admission job attachment status, pain intensity, and perceived disability. Given the substantial differences between employed and unemployed claimants further analyses were stratified based on admission job attached status while the other variables were controlled for in multivariable analysis. Intracluster kappa values for the RTW outcome across therapist clusters was small (Kappa = 0.01).

Table 2 presents a descriptive analysis of key claimant characteristics for injured workers who were non-job attached at time of referral to the RTW program (MI group = 125, control group = 74). Claimants were predominately male (80.4%), in their 40 s (average age 43 years), more likely to be single (33.7%) than married (26.1%), have a high school diploma, had an annual income of $69,600 CDN, with a disability duration of 481.8 days, and had moderate levels of pain and disability (mean pain VAS = 5.1/10 and mean Pain Disability Index = 52/70). No statistically significant differences were observed between non-job attached claimants in the MI and control groups on claimant characteristics such as sex, marital status, income, education level, age, disability duration, pain intensity or self-rated disability.

**Table 2:** Characteristics of non-job attached claimants at referral for return-to-work program

	Entire sample (n = 199)	Intervention group (n = 125)	Control group (n = 74)
	Mean (SD) or %		
Age (years)	43 (12.4)	43 (12.3)	43 (12.7)
Sex (% male)	80.4	78.4	83.8
Marital status (%)			
Married	26.1	25.6	27.0
Single	33.7	36.0	29.7
Common-law	13.1	12.8	13.5
Widowed	1.5	0.8	2.7
Divorced	8.5	10.4	5.4
Separated	6.5	4.8	9.5
Not specified	10.6	9.6	12.2
Gross annual salary	69.6 (35.4)	70.6 (34.8)	68.0 (36.6)
($10K CDN)			
Education level			
Grade 8 or less	6.0	7.2	4.1
Partial high school	15.6	18.4	10.8
High school diploma	25.1	23.2	28.4
Partial technical school	8.0	4.8	13.5
Technical diploma	20.1	24.8	12.2
Partial university	4.5	3.2	6.8
University degree	2.5	1.6	4.1
Not specified	18.1	16.8	20.3
Disability duration (days)	481.8 (1250.1)	471.9 (1170.0)	498.6 (1383.3)
Admission Pain Disability Index (PDI, n = 195)	52.0 (21.3)	53.2 (21.1)	49.9 (21.6)
Discharge PDI (n = 159)	43.2 (24.8)	42.1 (24.4)	45.4 (25.6)
Admission Visual Analogue Scale (VAS, n = 194)	5.1 (2.2)	5.2 (2.2)	4.9 (2.2)
Discharge VAS (n = 155)	4.5 (2.5)	4.4 (2.6)	4.6 (2.4)

There were no statistically significant differences observed between groups at p < 0.05

Table 3 presents a descriptive analysis of key claimant characteristics for injured workers who were job attached at the time of referral to the RTW program (MI group = 242, control group = 287). Claimants were predominately male (56.7%), in their 40 s (average age 45 years), more likely to be married (44.6%) than single (27.8%), have a high school diploma, had an annual income of $55,700 CDN, with a disability duration of 140.3 days, and had moderate levels of pain and disability (mean pain VAS = 5.0/10 and mean Pain Disability Index = 47/70). No statistically significant differences were observed between job-attached claimants in the intervention and control groups on claimant characteristics such as sex, marital status, income, education level, age, disability duration, pain intensity or self-rated disability.

**Table 3:** Characteristics of job attached claimants at referral for return-to-work program

	Entire sample (n = 529)	Intervention group (n = 242)	Control group (n = 287)
	Mean (SD) or %		
Age (years)	45 (12.0)	44 (11.8)	46 (12.2)
Sex (% male)	56.7	60.3	53.7
Marital status (%)			
Married	44.6	43.8	45.3
Single	27.8	27.3	28.2
Common-law	8.9	7.4	10.1
Widowed	1.3	1.7	1.0
Divorced	6.0	5.0	7.0
Separated	2.5	3.7	1.4
Not specified	8.9	11.2	7.0
Gross annual salary ($10 k CDN)	55.7 (26.7)	56.2 (26.9)	55.3 (26.7)
Education level			
Grade 8 or less	3.0	2.1	3.8
Partial high school	13.4	11.6	15.0
High school diploma	24.6	23.6	25.4
Partial technical school	9.6	10.7	8.7
Technical diploma	18.7	18.2	9.2
Partial university	3.6	3.7	3.5
University degree	8.7	9.1	8.4
Not specified	18.3	21.1	16
Disability duration (days)	140.3 (183.8)	135.3 (140.2)	146.2 (225.0)
Admission Pain Disability Index (PDI, n = 524)	47.0 (20.6)	47.1 (19.4)	47.0 (21.6)
Discharge PDI (n = 465)	31.5 (22.1)	32.1 (22.3)	31.1 (21.9)
Admission Visual Analogue Scale (VAS, n = 519)	5.0 (2.2)	5.0 (2.1)	4.9 (2.2)
Discharge VAS (n = 453)	3.6 (2.3)	3.6 (2.4)	3.5 (2.3)

There were no statistically significant differences observed between groups at p < 0.05

A higher percentage of claimants were observed to be male among claimants who are non-job attached in both the intervention and control groups (78.4 and 83.8%) compared to job attached claimants in the intervention and control groups (60.3 and 53.7%); however this was not statistically significant. Claimants who were non-job attached at program admission in both the intervention and control groups were more likely to be single (36 and 29.7%) while claimants who were job attached at program admission, in both the intervention and control groups, were more likely to be married (43.8 and 45.3%). Common law, divorced, and widowed responses were not considered as a part of the single or married categories. Claimants who were non-job attached at program admission in both the MI and control groups made +$10,000 more annually than claimants who were job attached at program admission despite group membership. High school diploma was the most common education level obtained in the MI and control groups for both non-job attached and job attached claimants. Disability duration was longer among the control groups for job attached and non-job attached claimants (146.2 and 498.6 days) compared to job attached and non-job attached claimants in the intervention groups (135.3 and 471.9 days); however, this was not statistically significant.

### Intervention Adherence

Table 4 presents documented MI adherence among intervention clinicians. Clinicians reported completing MI on between 7 and 79% of claimants and session durations lasted between 10 and 50 min. Four target behaviour categories were identified during the study; however RTW accounted for 75% of the target behaviours during the MI sessions. Due to the large difference in adherence rates among intervention clinicians, a sub analysis was completed to evaluate RTW percentages based on level of MI adherence (i.e. comparing the control, MI adherent, and non-adherent groups).

**Table 4:** Motivational Interviewing (MI) adherence among intervention clinicians

Clinician	Claimants during study (n)	Claimants MI completed with (%)	MI session duration (min)
1	72	5	10–30
2	71	56	15–20
3	65	8	10–30
4	64	17	10–45
5	47	4	15–50
6	48	6	30–40

Target behaviours			Target behaviour (%)	
RTW/finding new employment (i.e. modified work, vocational programs)			75	
Continue with/participate in RTW program i.e. lifting, educational workshops, individual counselling			18	
Make healthier choices (i.e. stop smoking, decrease medication use control blood pressure, deal with personal issues)			5	
Return to regular life/sport/physical activity			2	

### Program Outcomes (Proportion Successfully Returning to Work)

A chi square analysis was used to compare the proportion of successful RTW at program discharge between the intervention and control groups. Successful RTW at program discharge was 12.1% higher for unemployed claimants in the intervention group (intervention group 21.6 vs. 9.5% in control, p = 0.03) and 3.0% higher for job attached claimants compared to the control group (intervention group 97.1 vs. 94.1% in control, p = 0.10) (see Table 5).

**Table 5:** Program outcomes

Non-job attached			Program outcome		
			RTW	FFW	Total
MI intervention variable	Control group	Count	7	67	74
		%	9.5	90.5	100
	MI group	Count	27	98	125
		%	21.6*	78.4	100
Total		Count	34	165	199
		%	17.1	82.9	100

Job attached			Program outcome		
			RTW	FFW	Total
MI intervention variable	Control group	Count	270	17	287
		%	94.1	5.9	100
	MI group	Count	235	7	242
		%	97.1	2.9	100
Total		Count	505	24	529
		%	95.5	4.5	100

*Statistically significant difference on chi squared test (p = 0.03)

*MI* Motivational Interviewing, *RTW* return to work (i.e. confirmed outcome at program discharge that the worker is returning to work either at the pre-accident job or work with a new employer), *FFW* fit for work (i.e. worker has been deemed capable of working at program discharge but does not have a job to return to)

Statistically significant differences (p < 0.01) among RTW percentages were found for non-job attached claimants between MI adherent and non-adherent clinicians. The proportion of claimants with successful RTW in the MI adherent intervention group was 33.3%, which was significantly (p < 0.01) higher than the non-adherent intervention group (18.0%) and the control group (9.5%). Successful RTW percentage increased to 47.4% when the MI adherent intervention included RTW as the target behaviour. A statistically significant difference (p = 0.03) was also found among RTW for job attached claimants based on MI adherence, with RTW higher among the MI adherent group (100%) compared to the non-adherent MI group (96.3%) and the control group (94.1%).

### Multivariable Logistic Regression

Crude odds ratios (OR) for the intervention variable were 2.64 [95% confidence interval (CI) 1.09–6.41] in unemployed claimants and 2.11 (95% CI 0.86–5.19) in employed claimants. After adjusting for age, sex, annual salary, marital status, pain intensity, disability duration, and perceived disability, the OR for the intervention variable changed to 3.76 (95% CI 1.38–10.25) in unemployed claimants and 2.00 (95% CI 0.77–5.19) in employed claimants. The OR for unemployed claimants reduced to 2.64 (95% CI 0.69–10.14) and increased to 2.50 (95% CI 0.68–9.14) for employed claimants after adjusting for therapist cluster.

### Adverse or Unintended Effects

No negative or unintended effects were reported by the clinicians or claimants during the duration of the study. Stakeholders including case managers, physicians, and employers also did not report any adverse effects during the period of the study.

## Discussion

MI in addition to functional restoration programs appears to improve RTW outcomes among injured workers with musculoskeletal disorders, especially those admitted without jobs to return to. The use of MI likely helped some non-job attached claimants to resolve ambivalence regarding RTW. A smaller effect was observed for job-attached claimants, however, the overall proportion of successful RTW was substantially higher in this group with little room for additional improvement. Despite this, a statistically significant difference was observed when we accounted for MI adherence. The proportion of job-attached claimants with successful RTW was 100% in the intervention group when the MI protocol was adhered to, which was significantly higher than the control group (94%) and non-adherent group (96.3%). MI may, therefore, also have a role among claimants that are job attached given that the intervention is low cost and low risk [21]. The higher proportion of RTW in the MI group was observed despite no significant differences in pain or self-rated disability outcomes between groups, which is consistent with MI’s focus on changing a specific target behavioural goal (i.e. RTW). To our knowledge, this is the first evaluation of MI in the population of injured workers undergoing rehabilitation. Longer-term follow up of sustainable RTW between the intervention and control groups are required to determine the sustainability of the effects of MI.

Documented MI adherence among intervention clinicians varied substantially but were generally poor with only a 26% overall adherence rate. It may be that only some clinicians used MI but did not complete the adherence checklist, which was not part of routine paperwork. Alternatively, it may have been the case that some claimants were deemed inappropriate for MI or that some clinicians did not find MI useful, thus leading to low documented adherence rates. Regardless, we found that clinicians in the MI group with documented adherence had better RTW outcomes at program discharge especially if RTW was a target behaviour of MI. Future research should evaluate clinician’s beliefs and experiences regarding the benefits and challenges of integrating MI into functional restoration programs, as well as clinician or claimant characteristics leading to adherence and subsequent impact on outcomes. In addition, future research would likely benefit from added structure surrounding MI adherence to increase the fidelity of the intervention.

The applicability of MI across a variety of issues, its brief and specific interactions, and practical use in combination with other active treatment methods has contributed to the relevance of this intervention in work rehabilitation practice [27]. Results of this study will provide disability providers with information regarding the impact of MI on RTW, including statistical and clinical relevance. Future research should focus on the inclusion of all claimants who enter a RTW program in the compensation system, which could provide more insight into the application of MI in work rehabilitation for claimants with traumatic head or psychological injuries. Future studies should also consider evaluating stage-based interventions that could assist in increasing RTW among claimants who are at various levels of readiness for RTW.

There are some possible alternative explanations for our findings. Higher annual income levels were observed among non-job attached workers that attended the RTW program, which may be a contributing motivational factor in securing employment upon program discharge. In addition, claimants who were non-job attached were more likely to be single which may have resulted in the earlier identification of intrinsic motivational factors for RTW compared to those who are married and possibly turn to social supports, such as their spouses. However, the effect of our intervention variable was not confounded by the addition of salary or marital status to final models indicating these explanations are unlikely. Adjusting for therapist cluster did reduce the OR substantially, indicating that the effectiveness of MI may be differential across clinicians. However, the adjusted OR was still clinically meaningful (i.e. >2.0) after controlling for cluster effects.

Strengths of the study include a pragmatic clinical context using a cluster randomized controlled trial design. Data were gathered in a Canadian occupational rehabilitation setting as part of routine client care with relatively few restrictions placed on our sample. This should provide a fairly accurate representation of claimants in this Canadian Compensation system undergoing occupational rehabilitation. In addition, no adverse effects were reported as a result of this study by either clinicians or claimants.

Study limitations include the exclusion of claimants with brain injury and traumatic psychological injury. It is unknown how MI could potentially impact RTW within these specific subpopulations of injured workers, however these are typically complex cases that may benefit from additional RTW interventions aimed at overcoming psychosocial barriers to RTW. This study also did not include claimants who required the use of an interpreter during their RTW program due to the extensive linguistic component necessary for MI and the potential bias introduced by having an interpreter translate for the claimant. Another limitation is the unequal number of job attached claimants between the intervention and control groups. At the occupational rehabilitation facility where the data were collected, there are three potential RTW programs the claimants were triaged to and one of these excluded non-job attached claimants. Both clinicians in this program who were involved in the study were randomized to the control group, leading to increased numbers of non-job attached claimants in the intervention group. This was not foreseen at the time of randomization, and we did not want to affect the randomization procedure after data collection had begun. Another limitation was the fairly low documented adherence to the MI intervention (full adherence reported in only 26% of cases). However, we observed that claimants with clinicians reporting full adherence had better claims outcomes than either the non-adherent or control group in the follow-up year. Future research is needed to evaluate the influence of therapist factors such as age, sex (including the dynamic between clinicians treating opposite-sex clients), training, and other relevant variables on adherence to MI protocols and subsequent clinical outcomes.

## Conclusion

MI integrated into work rehabilitation appears to be more effective than routine rehabilitation programs alone in improving RTW among workers with disabling MSK disorders. MI could be an important addition to work rehabilitation programs as there are currently few evidence based, non-physical intervention methods to address psychosocial and behavioural barriers to recovery associated with MSK disorders. Adherence to the MI protocol was mixed but RTW outcomes were substantially higher among MI adherent clinicians. Further research is needed to examine long-term sustainability of these outcomes, stage-based approaches to behavioural change through MI, as well as the varied clinician adherence and its effect on outcomes.
